# The G-Protein-Coupled Bile Acid Receptor Gpbar1 (TGR5) Inhibits Gastric Inflammation Through Antagonizing NF-κB Signaling Pathway

**DOI:** 10.3389/fphar.2015.00287

**Published:** 2015-12-11

**Authors:** Cong Guo, Hui Qi, Yingjie Yu, Qiqi Zhang, Jia Su, Donna Yu, Wendong Huang, Wei-Dong Chen, Yan-Dong Wang

**Affiliations:** ^1^State Key Laboratory of Chemical Resource Engineering, College of Life Science and Technology, Beijing University of Chemical TechnologyBeijing, China; ^2^Key Laboratory of Receptors-Mediated Gene Regulation and Drug Discovery, School of Medicine, Henan UniversityKaifeng, China; ^3^Key Laboratory of Molecular Pathology, School of Basic Medical Science, Inner Mongolia Medical UniversityHohhot, China; ^4^Department of Materials Science and Engineering, State University of New York at Stony BrookStony Brook, NY, USA; ^5^Department of Diabetes and Metabolic Diseases Research, Beckman Research Institute, City of Hope National Medical CenterDuarte, CA, USA

**Keywords:** tgr5, GPBAR1, GPCR, NF-κB, gastric inflammation

## Abstract

Gpbar1 (TGR5), a membrane-bound bile acid receptor, is well-known for its roles in regulation of energy homeostasis and glucose metabolism. Here, we show that mice lacking TGR5 were much more susceptible to lipopolysaccharide (LPS)-induced acute gastric inflammation than wild-type (WT) mice and TGR5 is a negative regulator of gastric inflammation through antagonizing NF-κB signaling pathway. We found that the treatment of TGR5 ligands 23(S)-mCDCA and GPBARA (3-(2-Chlorophenyl)-N-(4-chlorophenyl)-N,5-dimethylisoxazole-4-carboxamide) suppressed gene and protein expression mediated by NF-κB signaling. TGR5 overexpression with ligand treatment inhibited gene expression of interferon-inducible protein 10 (IP-10), TNF-α, and chemoattractant protein-1 (MCP-1) induced by LPS. Furthermore, we revealed that TGR5 activation antagonized NF-κB signaling pathway through suppressing its transcription activity, the phosphorylation of IκBα and p65 translocation, which suggests that TGR5 antagonizes gastric inflammation at least in part by inhibiting NF-κB signaling. These findings identify TGR5 as a negative mediator of gastric inflammation that may serve as an attractive therapeutic tool for human gastric inflammation and cancer.

## Introduction

Chronic inflammation is increasingly recognized as an important tumor promoter (Pikarsky et al., 2004; Yoshizaki et al., 2010). The precise control of inflammation is essential for the prevention of chronic inflammatory disorders, as well as for inhibiting the exacerbation or progression of diseases, including many types of cancers. Gastric cancer is an inflammation-associated cancer because *Helicobacter pylori*, which infects 50% of the world's population, is now known to be responsible for inducing chronic gastric inflammation that progresses to atrophy, metaplasia, dysplasia, and gastric cancer (Fox and Wang, 2007). Thus, the control of gastric inflammation is important for the prevention and treatment of gastric cancer (Karin and Greten, 2005; Hotamisligil, 2008).

Activated NF-κB is frequently detected in various inflammatory diseases and tumors. The activation of NF-κB is one of the critical cellular responses to acute infections and inflammations (Aggarwal, 2004; Karin and Greten, 2005). So NF-κB has received extensive attention as a key regulator of inflammation and carcinogenesis (Pikarsky et al., 2004; Fox and Wang, 2007). In response to lipopolysacchride (LPS) or pro-inflammatory cytokines, NF-κB can be rapidly activated. Pikarsky et al. and Greten et al. reported that the classical, IKK-dependent NF-κB-activation pathway is a crucial mediator of tumor progression (Greten et al., 2004; Pikarsky et al., 2004). The classic form of NF-κB is the heterodimer of the p65/RelA and p50 subunits. It is activated in response to various stimuli, including LPS, TNF-α, double-stranded RNA, and ultra-violet radiation. Under normal conditions, NF-κB signaling are tightly controlled by multiple negative feedback mechanisms. Conversely, chronic activation of NF-κB signaling is frequently detected in numerous human inflammatory diseases and cancer, including gastric tumorigenesis (Hedvat et al., 2009; Lu et al., 2014; Yang et al., 2015). Thus, constitutive NF-κB activation is fundamental to the pathobiology of gastric cancer (D'Acquisto and Ianaro, 2006; Lu et al., 2014). Therefore, defining new therapeutic targets that inhibit prolonged activation of NF-κB signaling is crucial for further understanding the regulation of this signaling pathway and the development of novel therapeutic strategies to improve disease symptoms in gastric inflammation and cancer (Israel et al., 2001; Fox and Wang, 2007).

TGR5, as a bile acid membrane receptor, can regulate bile acid homeostasis, energy homeostasis, and glucose metabolism (Kawamata et al., 2003). It belongs to a member of the G-protein-coupled receptor (GPCR) family which contains seven transmembrane domains and transduces extracellular signals through heterotrimeric G proteins (Duboc et al., 2014). We and other group reported that TGR5 is a negative modulator of NF-κB-mediated liver inflammation. TGR5 activation suppressed NF-κB-mediated liver inflammation through inhibiting phosphorylation of IκBa and nuclear translocation of p65 *in vitro* and *in vivo* (Hedvat et al., 2009; Wang et al., 2011). *Helicobacter pylori* infection upregulates NF-κB to induce inflammation in the stomach (Yang et al., 2012). Chronic inflammation is a frequent cause of cancer (Fox and Wang, 2007; Zhang et al., 2014). Disrupting the aberrant activation of NF-κB signaling is able to dramatically suppress tumor progression (Lu et al., 2014). Therefore, the previous results raise the possibility that TGR5 may be a negative regulator of gastric inflammation possibly through antagonizing NF-κB signaling in stomach.

In this study, we show that TGR5 activation suppresses LPS-induced gastric inflammation *in vitro* and *in vivo*. Furthermore, we identified that TGR5 is a negative regulator of NF-κB signaling pathways in gastric cancer cells via suppressing its transcription activity, the phosphorylation of IκBα and p65 translocation, respectively. These findings suggest TGR5 may be a potential target for therapeutic intervention in human gastric inflammation through antagonizing NF-κB signaling.

## Materials and methods

### Reagents and plasmids

Lipopolysaccharide (LPS, from Escherichia *coli* 0111:B4) was purchased from Sigma Chemical (St. Louis, MO). TGR5 ligand 23(S)-mCDCA was provided by Dr. Wendong Huang and Dr. Donna Yu (City of Hope, Duarte, CA). 23(S)-mCDCA is a synthetic, highly selective TGR5 agonist used in the previous work (Pellicciari et al., 2007; Wang et al., 2011). GPBARA [TGR5 Receptor Agonist, 3-(2-Chlorophenyl)-N-(4-chlorophenyl)-N,5-dimethylisoxazole-4-carboxamide] has been used in the previous reports (Inoue et al., 2012; Jensen et al., 2013). It was purchased from BioVision (Milpitas, CA). The pmTGR5 expression vector was created in our laboratory. The p65 expression vector and the phRL-TK vector were kindly provided by Xufeng Chen and Akio Kruoda (both City of Hope, Duarte, CA), respectively. The NF-κB-dependent reporter (NF-κBx3-LUC) was provided by Dr. Peter Tontonoz (UCLA, Los Angeles, CA) and Dr. Bruce Blumberg (UCLA, Los Angeles, CA).

### Animals

Eight-week-old wild-type (WT) (C57BL/6J) and TGR5^−∕−^ female mice (on C57BL/6J background; Merck Research Laboratories, Kenilworth, NJ) were maintained in a pathogen-free animal facility under a standard 12-h light-dark cycle. In the preliminary study, we screened the doses of TGR5 ligand 23(S)-mCDCA for *in vivo* use. It was found that diet containing 10 mg/kg of 23(S)-mCDCA was an optimal dose. So mice were fed a diet containing 10 mg of 23(S)-mCDCA/kg diet or standard rodent chow for 3 days. After that, mice were fasted overnight and then injected intraperitoneally (i.p.) with a single dose of LPS (20 mg/kg) or phosphate-buffered saline (PBS), followed by feeding water *ad libitum*. Six hours after the injection, mice were killed by CO_2_ asphyxiation, and the stomach was removed for further analysis. The animal study proposal was approved by Beckman Research Institute of City of Hope Institutional Animal Care and Use Committee (IACUC). All animal experiments were carried out in accordance with an approved Beckman Research Institute of City of Hope Institutional Animal Care and Use Committee (IACUC) protocol.

### Cell culture and transfection

Gastric cancer cell line SGC7901 was obtained from Institute of Basic Medical Sciences (IBMS) of Chinese Academy of Medical Sciences. Cells were grown in complete culture medium (RPMI-1640 [with L-glutamihe] supplied with 10% (vol/vol) inactivated fetal calf serum and 1% (vol/vol) antibiotics-antimycotics). Cultures were fed with fresh medium twice weekly. For experiments, 6 × 10^5^ SGC7901 cells were seeded in 60-mm culture dishes with complete culture medium. Transient transfection of SGC7901 cells with TGR5 expression plasmid was performed using Lipofectamine 2000 (Invitrogen, Carlsbad, CA). Twenty-four hours after transfection, cells were pre-treated with 23(S)-mCDCA (10 μM) or GPBARA (3 μM) for 1 day. Then cells were treated with or without LPS or TNF-α. Following a 6-h incubation for LPS or a 1-h incubation for TNF-α, cells were harvested for Quantitative Real-Time PCR analysis. For protein assay, cells were transfected with TGR5 plasmid and then pre-treated with 23(S)-mCDCA (10 μM) or GPBARA (3 μM) for 1 day. Then cells were treated with TNF-α (10 ng/mL) for 1 h. Finally, cells were collected for total protein isolation and Western blot assay. For luciferase assay, transient transfection of SGC7901 cells with the NF-κB reporter plasmid, phRL-TK, and/or TGR5 expression plasmid was performed. Twenty-four hours after transfection, cells were pre-treated with 23(S)-mCDCA (10 μM), GPBARA (3 μM) or vehicle (dimethyl sulfoxide (DMSO)) for 24 h. Then cells were treated with/without LPS (20 μg/mL) or TNF-α (10 ng/mL). After 6 h of incubation, cells were harvested and the luciferase activity was determined using a dual-luciferase reporter assay system in accordance with the manufacturer's instructions (Promega, Madison, WI). Luciferase activities were normalized by co-transfection of the control thymidine kinase-driven Renilla luciferase plasmid, phRL-TK. Data are expressed as relative fold activation to that of non-stimulated (–) sets.

### RNA isolation and quantitative real-time polymerase chain reaction

Total RNA was extracted from SGC7901 cells using Tri-Reagent (Molecular Research Center, Inc., Cincinnati, OH). Quantitative real-time PCR was performed using the Power SYBR Green PCR Master Mix protocol (Applied Biosystems, Foster City, CA). Amplification of β-actin was used as an internal reference. β-Actin primers were obtained from Ambion, Inc. (Austin, TX). Quantitative PCR analysis was conducted using the ABI 7300 Sequence Detection System. Primers sequences are available on request.

### Immunoblot analysis

At indicated time points after treatment, SGC7901 cells were lysed for 30 min with lysis buffer and centrifuged at 12,000 × g at 4°C for 15 min. The samples were resolved by 10% sodium dodecyl sulfate–polyacrylamide gel electrophoresis, transferred to nitrocellulose membranes, and blotted using primary antibodies. The membranes were washed with Tris Buffered Saline with 0.1% Tween® 20 (TBST) and then incubated with anti-rabbit secondary antibody conjugated to horseradish peroxidase (HRP) (1:5000) (Thermo Scientific, Waltham, MA). Bands on blots were visualized using Tanon 5200 enhanced chemiluminescence (ECL) detection system (Tanon, China) and quantified with a computerized digital imaging system using Tanon software.

### Enzyme-linked immunosorbent assay (ELISA)

Mice were fed a diet containing 10 mg of 23(S)-mCDCA/kg diet or standard rodent chow for 3 days. After that, mice were fasted overnight and then injected intraperitoneally (i.p.) with a single dose of LPS (20 mg/kg) or phosphate-buffered saline (PBS), followed by feeding water *ad libitum*. Six hours after the injection, mice were killed, and the stomach was removed for further protein analysis. Stomach proteins were extracted in cold PBS and MCP-1 and IP-10 protein levels were determined using Enzyme-linked Immunosorbent Assay (ELISA) Kit in accordance with the manufacturer's instructions (Cloud-Clone Corp., Houston, TX). For cell culture, the cells were treated with the indicated reagents. Then proteins were extracted with cold PBS and determined using ELISA kit in accordance with the manufacturer's instructions (Cloud-Clone Corp., Houston, TX).

### Statistics

All data represent at least three independent experiments and are expressed as the mean ± SD. The Two-way analysis of variance (ANOVA), followed by Bonferroni's *post-hoc* test, was performed. A *P* < 0.05 was considered significant.

## Results

### TGR5^−∕−^ mouse stomach displays elevated expression of proinflammatory genes

**Figure 1:**
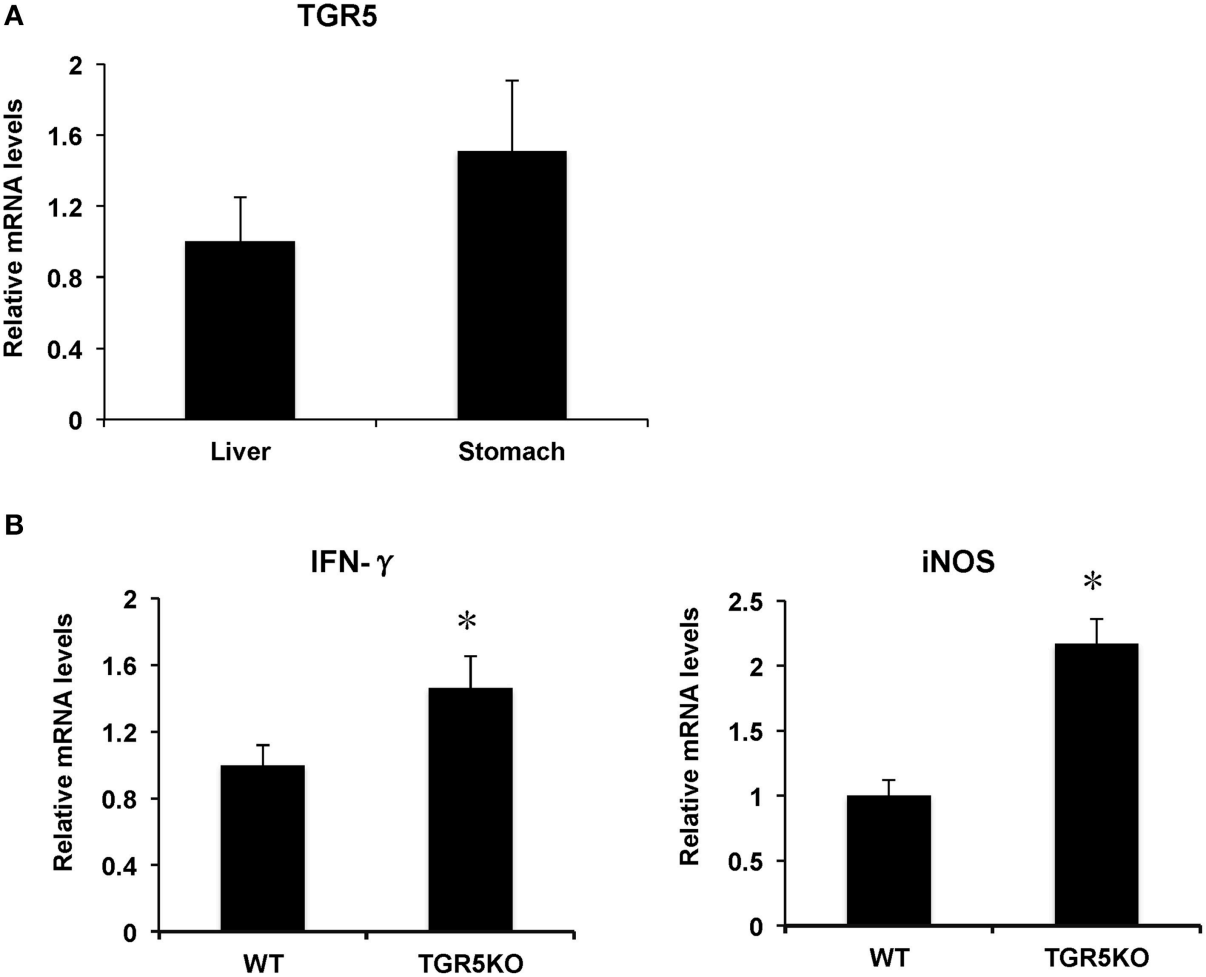
**TGR5 is expressed in stomach and TGR5 ^−∕−^ mouse stomach displays elevated expression of proinflammatory genes**. **(A)** Levels of TGR5 gene expression in mouse stomach and liver (*n* = 5). **(B)** TGR5^−∕−^ mouse stomach display elevated expression of proinflammatory genes compared with WT mice (*n* = 5). ^*^P < 0.05 vs. WT mice. TGR5KO, TGR5^−∕−^ mice.

TGR5 is expressed in many organs such as liver, colon, small intestine, kidney, heart, and stomach. In this work, we found that TGR5 gene is expressed in stomach slightly higher than that in liver (Figure 1A). Compared with WT controls, stomach from TGR5^−∕−^ mice had elevated messenger RNA (mRNA) levels of some proinflammatory genes (Figure 1B). These elevated genes include interferon-γ (IFN-γ) and inducible nitric oxide synthase (iNOS).

### TGR5 activation suppresses gastric inflammation *in vivo*

**Figure 2:**
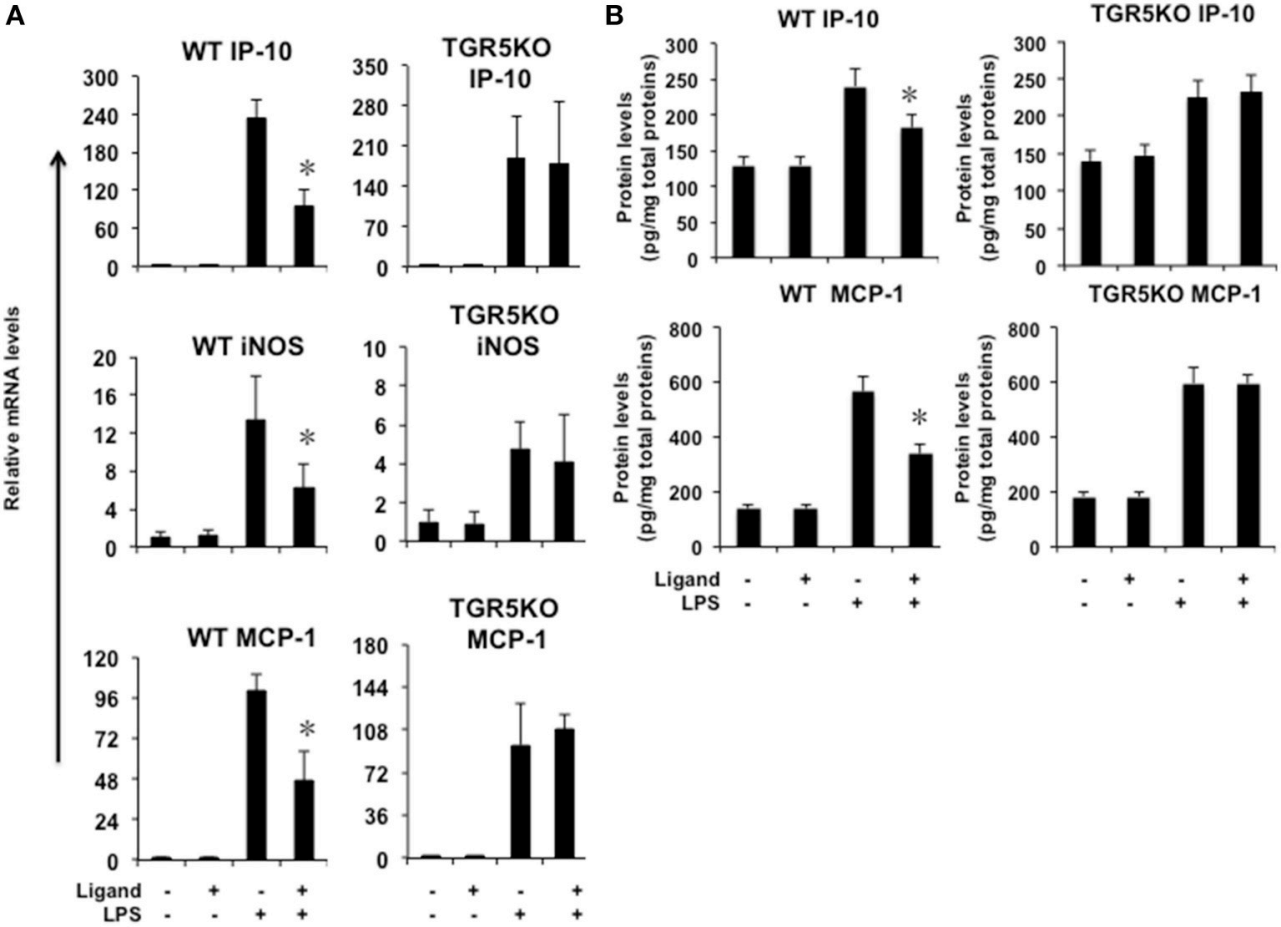
**TGR5 activation suppresses gastric inflammation ***in vivo*****. Mice were fed a diet containing 10 mg of TGR5 ligand 23(S)-mCDCA/kg diet or standard rodent chow for 3 days. After that, mice were fasted overnight and then injected intraperitoneally (i.p.) with a single dose of LPS (20 mg/kg) or phosphate-buffered saline (PBS). Six hours after the injection, mice were sacrificed and the stomach was removed for Real-Time PCR analysis or ELISA analysis. Ligand, 23(S)-mCDCA. **(A)** TGR5 ligand 23(S)-mCDCA treatment repressed LPS-induced proinflammatory gene expression in WT, but not TGR5^−∕−^ mouse stomach (*n* = 5–6). ^*^P < 0.05 vs. the only LPS-treated WT groups. **(B)** TGR5 ligand 23(S)-mCDCA treatment repressed LPS-induced MCP-1 and IP-10 protein expression in WT, but not TGR5^−∕−^ mouse stomach (*n* = 5–6). ^*^P < 0.05 vs. the only LPS-treated WT groups.

If TGR5 is a suppressor of gastric inflammation, TGR5 activation may suppress some proinflammatory gene expression. We firstly tested whether ligand-activated TGR5 could inhibit NF-κB-mediated proinflammatory genes *in vivo*. TGR5 activation by 23(S)m-CDCA repressed LPS-induced interferon-inducible protein 10 (IP-10), iNOS and monocyte chemoattractant protein-1 (MCP-1) gene expression in WT stomach, but not TGR5^−∕−^ stomach (Figure 2A). Protein levels of MCP-1 and IP-10 in WT and TGR5^−∕−^ mouse stomach were also tested using ELISA (Figure 2B). The results show that TGR5 activation by 23(S)m-CDCA repressed LPS-induced IP-10 and MCP-1 protein expression in WT stomach, but not TGR5^−∕−^ stomach (Figure 2B).

### Activation of TGR5 antagonizes NF-κB-mediated gene expression in gastric cancer cells

**Figure 3:**
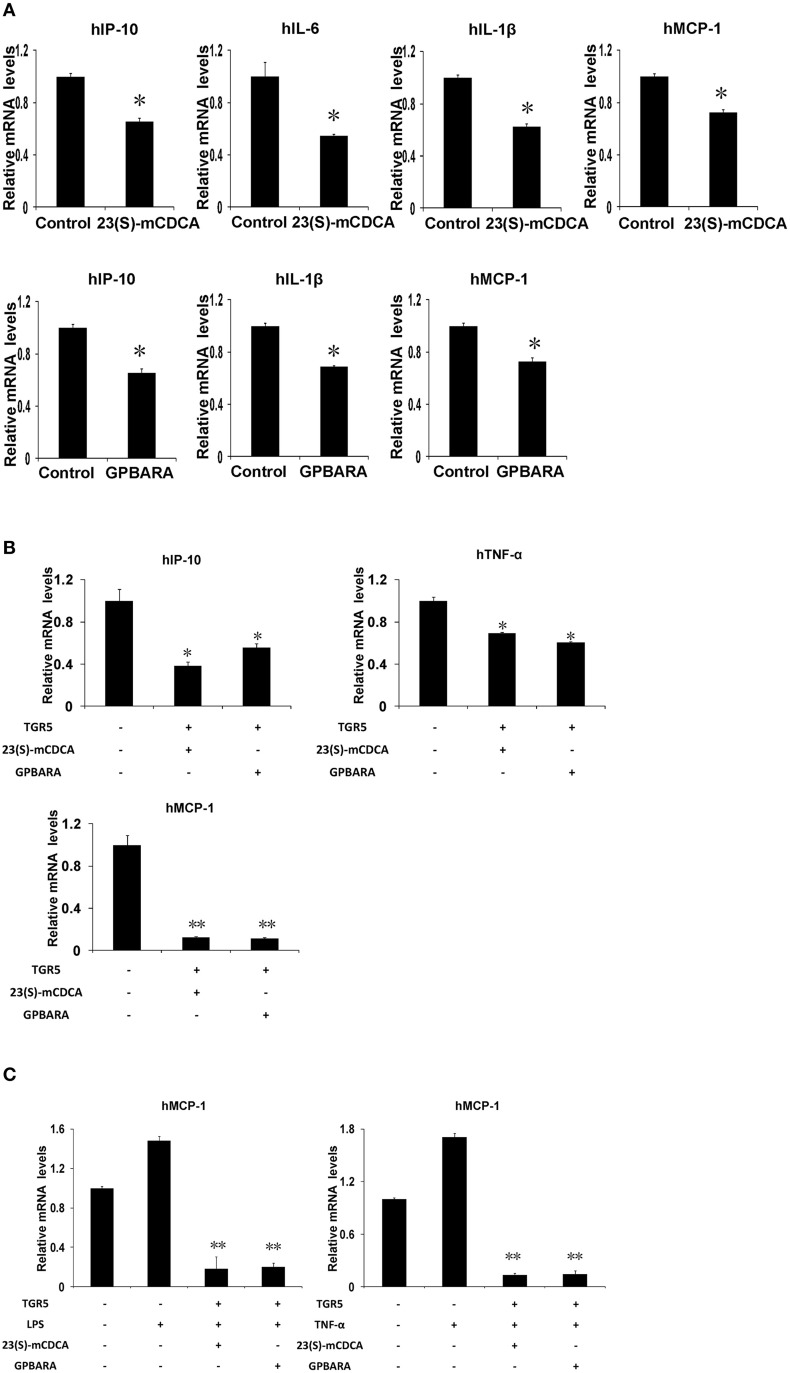
**Activation of TGR5 antagonizes NF-κB-mediated gene expression in gastric cancer cells**. **(A)** TGR5 ligand treatment suppresses NF-κB-mediated gene expression. 23(S)-mCDCA and GPBARA treated SGC7901 cells for 24 h. **(B)** TGR5 overexpression with ligand treatment suppresses NF-κB-mediated gene expression. SGC7901 cells were transfected with the TGR5 expression plasmid or control plasmid. After transfection, cells were treated with GPBARA (3 μM), 23(S)-mCDCA (10 μM), or vehicle (DMSO) for 24 h. **(C)** TGR5 activation suppresses LPS or TNF-α-induced gene expression. SGC7901 cells were transfected with the TGR5 expression plasmid or control plasmid. After transfection, cells were treated with GPBARA (3 μM) or vehicle (DMSO) for 24 h. Then cells were treated with LPS (10 μg/mL) for 6 h or TNF-α (10 μg/mL) for 1 h. ^*^P < 0.05, ^**^P < 0.005 vs. the control group. (*n* = 3).

Our previous work has indicated that TGR5 activation suppresses NF-κB-mediated gene expression in hepatocytes (Wang et al., 2011). To investigate whether activation of TGR5 has any effect on the NF-κB pathway in gastric cells, we tested the influence of TGR5 agonists on the NF-κB-mediated gene expression in SGC7901 gastric cancer cells. We firstly tested whether ligand-activated TGR5 could inhibit NF-κB-mediated gene expression. In SGC7901 gastric cancer cells, TGR5 ligand 23(S)-mCDCA suppresses gene expression of IP-10, interleukin (IL)-6, IL-1β, and MCP-1 mediated by NF-κB while GPBARA treatment suppresses gene expression of IP-10, IL-1β, and MCP-1 mediated by NF-κB (Figure 3A). TGR5 overexpression with the ligands 23(S)-mCDCA or GPBARA treatment repressed gene expression of IP-10, TNF-α, and MCP-1 mediated by NF-κB (Figure 3B). Furthermore, TGR5 activation suppressed LPS or TNF-α-induced MCP-1 expression (Figure 3C). Some of the results were also confirmed using ELISA assay to reveal TGR5 activation suppressed MCP-1 and IP-10 protein expression in gastric cancer cells (Figure S1 in Supplementary Material).

### Activation of TGR5 antagonizes NF-κB transcriptional activity in gastric cancer cells

**Figure 4:**
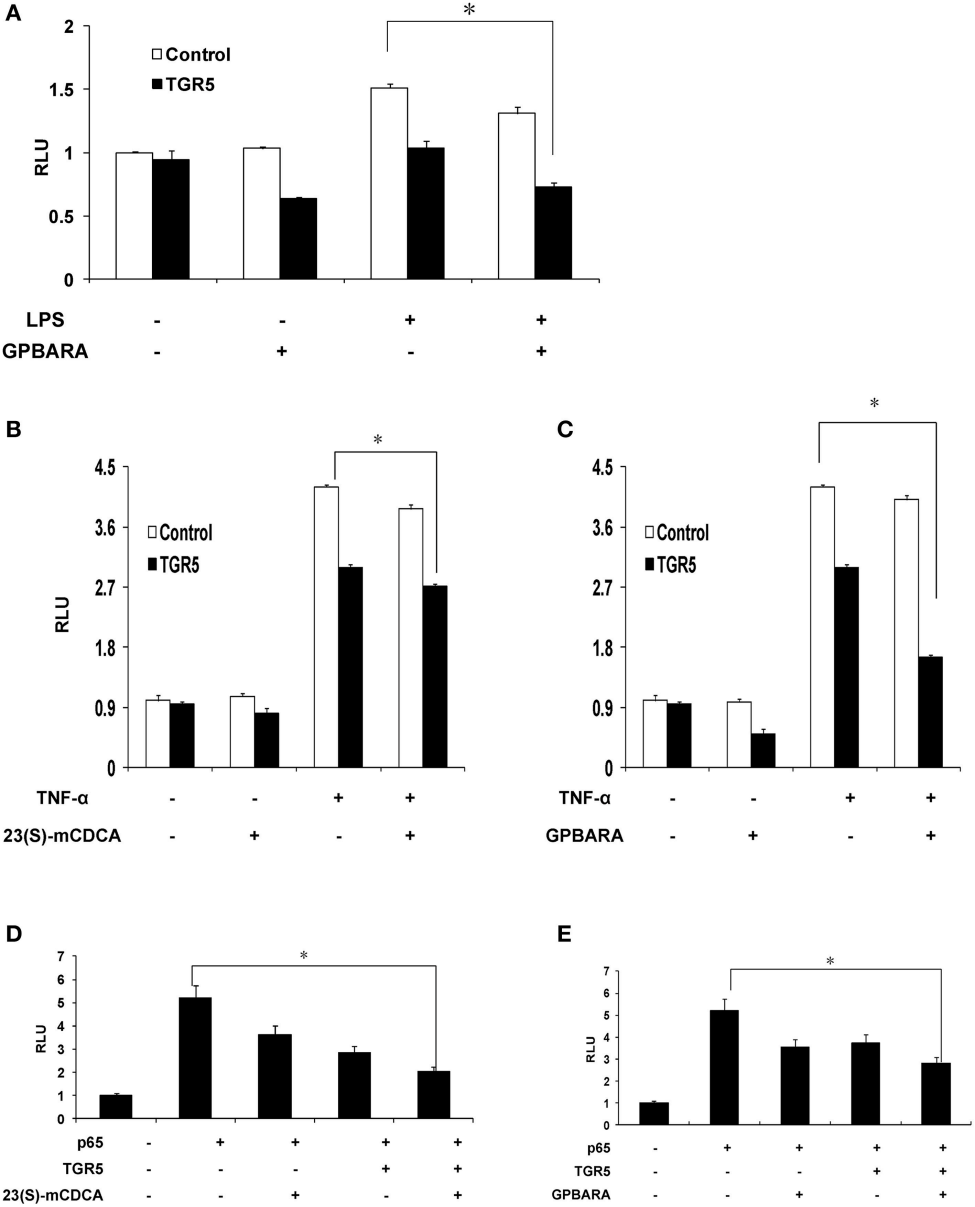
**Activation of TGR5 antagonizes NF-κB transactivity**. **(A)** TGR5 suppressed NF-κB transactivity induced by LPS. SGC7901 cells were cotransfected with the NF-κB reporter plasmid (pNF-κB-LUC), phRL-TK, and TGR5 expression plasmid. After transfection, cells were treated with GPBARA (3 μM) or vehicle (DMSO) for 24 h and then treated with LPS (20 μg/mL) for 6 h. **(B)** TGR5 ligand 23(S)-mCDCA suppressed NF-κB transactivity induced by TNF-α (10 μg/mL) for 6 h. **(C)** TGR5 ligand GPBARA suppressed NF-κB transactivity induced by TNF-α (10 μg/mL) for 6 h. **(D)** TGR5 ligand 23(S)-mCDCA suppressed NF-κB transactivity induced by p65 overexpression. **(E)** TGR5 ligand GPBARA suppressed NF-κB transactivity induced by p65 overexpression. ^*^P < 0.05. RLU, relative luciferase units (*n* = 3).

Because TGR5 activation by 23(s)m-CDCA and GPBARA inhibited the expression of NF-κB target genes, we next tested whether TGR5 activation inhibited NF-κB activity at the level of gene transcription. We cotransfected SGC7901 cells with an NF-κB reporter plasmid and the control plasmid phRL-TK and assessed the effects of GPBARA on the regulation of NF-κB reporter activity. Treatment with a known NF-κB pathway activator LPS resulted in 1.5-fold greater NF-κB reporter activity (Figure 4A). NF-κB activity induced by LPS was inhibited by GPBARA treatment. Transfection of these cells with TGR5 inhibited NF-κB activity in the absence of ligand. However, addition of GPBARA further enhanced this repression (Figure 4A). Furthermore, we used TNF-α to induce NF-κB reporter activity. TNF-α resulted in 4.2-fold greater NF-κB reporter activity (Figures 4B,C). TGR5 overexpression with 23(S)-mCDCA or GPBARA represses TNF-α-induced NF-κB reporter activity by about 35% (Figure 4B) and 60%, respectively (Figure 4C). To eliminate the possibility that the compound was affecting other pathways, we used p65 overexpression to activate the NF-κB reporter. Overexpression of p65 significantly activated the NF-κB reporter (Figures 4D,E). NF-κB activity was inhibited by both TGR5 ligands in the absence of TGR5, but the presence of TGR5 enhanced this repression.

### TGR5 inhibits IκBα phosphorylation and p65 translocation in gastric cancer cells

**Figure 5:**
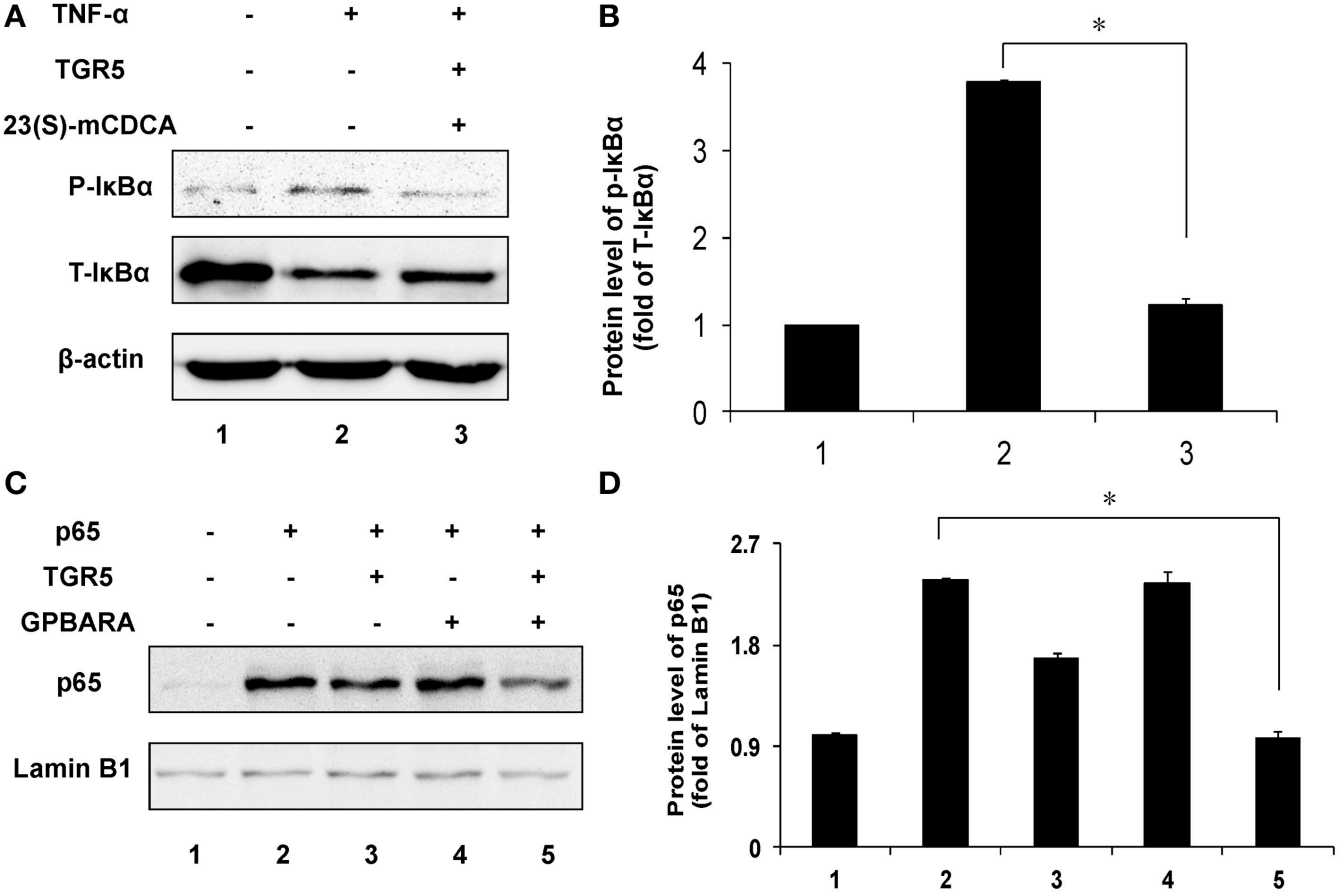
**TGR5 inhibits IκBα phosphorylation and p65 translocation in gastric cancer cells**. **(A)** TGR5 overexpression with ligand treatment suppressed TNF-α-induced phosphorylation of IκBα in SGC7901 cells. Cells were transfected with TGR5 plasmid and then treated with ligand for 24 h. Finally, cells were treated with TNF-α for 1 h (*n* = 3) p-IκBα, phosphorylated IκBα; T-IκBα, total IκBα. **(B)** The data of relative protein levels in **(A)** are expressed as fold change over the ratio of p-IκBα to T-IκBα in the control group (lane 1). **(C)** TGR5 overexpression with ligand treatment suppressed p65 overexpression-induced the translocation of p65 in SGC7901 cells. Cells were cotransfected with TGR5 and/or p65 plasmids and then were treated with the ligand GPBARA for 24 h. **(D)** The data of relative protein levels in (C) are expressed as fold change over the ratio of p65 to Lamin B1 in the control group (lane 1). ^*^P < 0.05.

Next, we tested the suppression of TGR5 activation on phosphorylation of IκBα. Compared with the control group, TNF-α induced phosphorylation of IκBα in SGC7901 cancer cells in a time-dependent manner (Figure S2A in Supplementary Material). TGR5-transfected SGC7901 cells with ligand treatment (23(S)-mCDCA) inhibited TNF-α-induced IκBα phosphorylation by about 65% (Figures 5A,B). The nuclear translocation of p65 leads to activation of NF-κB. Overexpression of p65 induced the translocation of p65 in a time-dependent manner (Figure S2B in Supplementary Material). TGR5 activation by GPBARA dramatically suppressed the nuclear translocation of p65 induced by p65 overexpression in gastric cancer cells (Figures 5C,D). These results demonstrated that TGR5 activation is able to suppress IκBα phosphorylation and nuclear translocation of p65.

## Discussion

The known functions of TGR5 *in vivo* have recently expanded rapidly from initial roles in regulating energy homeostasis and metabolic diseases to also participating in inflammation and carcinogenesis (Cipriani et al., 2011; Pols et al., 2011; Wang et al., 2011; Cao et al., 2013; Guo et al., 2015). The novel roles of TGR5 in suppressing inflammation are consistent with TGR5's previous roles in defending against diabetes and obesity. In contrast to its well-established mechanism in regulating glucose and energy homeostasis, little is known about how TGR5 functions in gastric inflammation and carcinogenesis. Our results suggest that one potential role for TGR5 in protecting against gastric inflammation is by modulating NF-κB-mediated gastric inflammatory responses. TGR5 activation strongly suppresses the activity of NF-κB in gastric cell culture experiments *in vitro*. This is further supported by animal studies *in vivo*.

TGR5 belongs to GPCR family (Wang et al., 2011). GPCRs play a crucial role in physiology and pathophysiology in humans through regulating cell migration, proliferation, differentiation and survival. They are very promising targets for the development of drugs having therapeutical impact on many diseases such as chronic inflammation, neurodegeneration, diabetes, stress, and osteoporosis (Couvineau and Laburthe, 2012; Saxena et al., 2012). Many GPCRs induce NF-κB activation (Islam et al., 2013), whereas only a few GPCRs inhibit NF-κB-mediated inflammation (Linden, 2006). Two GPCRs, the A2A and A2B adenosine receptors, suppress the NF-κB pathway in a specific gene- and cell-type–dependent manner (Lappas et al., 2005; Linden, 2006; Sun et al., 2006). Activation of β2-adrenergic receptor, a subtype of GPCRs, inhibits NF-κB activity by means of β-arrestin interaction with IκBα. Our previous data show that TGR5 is a potential suppressor of NF-κB-dependent inflammatory response through regulating interaction of β-arrestin2 and IκBα in liver inflammation (Wang et al., 2011). Other groups also reported that TGR5 activation is associated with different inflammation (Cipriani et al., 2011; Pols et al., 2011). Here, we found that TGR5 activation inhibited gastric inflammation. Furthermore, it is found that TGR5 activation antagonizes NF-κB signaling in gastric cancer cells through inhibiting its transcriptional activity, phosphorylation of IκBα and p65 translocation. These results suggest that TGR5 is a suppressor of gastric inflammation through antagonizing NF-κB signaling. It indicates that TGR5 has much broader role than previously thought in suppressing inflammation.

We noted that activation of TGR5 repressed specific sets of NF-κB target genes, but not all the target genes in response to the NF-κB activators that we used in this study (LPS, and TNF-α). This phenomenon has also been observed for the function of TGR5 in liver inflammation (Wang et al., 2011). Similar results obtained indicate the molecular mechanisms by which TGR5 suppressed NF-κB in liver and gastric inflammation may be similar. It would be interesting to define the mechanism by which TGR5 activation inhibits NF-κB in gastric cancer cells.

It is noted that transfection of gastric cancer cells with TGR5 inhibited NF-κB activity in the absence of ligand, suggesting that TGR5 may suppress NF-κB activity without the addition of exogenous ligand, possibly resulting from the fact that GPCRs have constitutive activity as previously reported (Tao, 2008; Senft et al., 2011; Wang et al., 2011).

It has been reported that TGR5 could be a potential target for the treatment of diabesity and associated metabolic disorders (Watanabe et al., 2006; Thomas et al., 2008). For example, Watanabe et al. reported that TGR5 activation by bile acids induces energy expenditure in muscle and brown adipose tissue (Watanabe et al., 2006). Thomas et al. found that TGR5 activation improves glucose tolerance and insulin sensitivity in fat-fed mice (Thomas et al., 2008). These diseases, such as obesity, insulin resistance, and type 2 diabetes, are also closely associated with chronic inflammation characterized by abnormal cytokine production, increased acute-phase reactants, and activation of a network of inflammatory signaling pathways (Hotamisligil, 2008). Combining with our previous study (Wang et al., 2011), our results show that TGR5 is a negative modulator of gastric and liver carcinogenesis probably by antagonizing NF-κB pathway. Therefore, there is a potential link between anti-cancer and treatment of obesity and diabetes through TGR5. TGR5 may be an attractive therapeutic target not only for metabolic disorders but also for cancer.

In conclusion, our results reveal that TGR5 is a suppressor of gastric inflammation and TGR5 activation suppresses NF-κB signaling pathway, indicating that TGR5 ligands have utility in anti-gastric inflammation. These findings suggest that TGR5 is a potential target for anti-inflammatory drug design, and its agonist ligands offer possible therapies to prevent and treat inflammatory gastric diseases.

## Funding

This work is supported by the National Natural Science Foundation of China (Grant No. 81370537) and the Fundamental Research Funds for the Central Universities (Grant No. YS1407 and 2050205) to YW, the National Natural Science Foundation of China (Grant No. 81270522 and Grant No. 81472232), Program for Science and Technology Innovation Talents in Universities of Henan Province (HASTIT, Grant No. 13HASTIT024), and Plan for Scientific Innovation Talent of Henan Province to WC.

### Conflict of interest statement

The authors declare that the research was conducted in the absence of any commercial or financial relationships that could be construed as a potential conflict of interest.
