# Supplementary material for: The G-Protein-Coupled Bile Acid Receptor Gpbar1 (TGR5) Inhibits Gastric Inflammation Through Antagonizing NF-κB Signaling Pathway
Source: Front Pharmacol. 2015 Dec 11;6:287. doi: 10.3389/fphar.2015.00287 (PMC4675858; doi:10.3389/fphar.2015.00287)
Supplement: Supplementary file 1 [file DataSheet1.DOCX]

**Supplementary Material**

**The G-protein-coupled bile acid receptor Gpbar1 (TGR5) inhibits gastric inflammation through antagonizing NF-κB signaling pathway**

Cong Guo,^1^ Hui Qi,^2,3†^ Yingjie Yu,^4†^ Qiqi Zhang,^1^ Jia Su,^1^ Donna Yu,^5^ Wendong Huang,^5^ Wei-Dong Chen,^2,3*^ Yan-Dong Wang^1*^

† Hui Qi and Yingjie Yu contributed equally to this work.

^1^ State Key Laboratory of Chemical Resource Engineering, College of Life Science and Technology, Beijing University of Chemical Technology, Beijing, P. R. China

^2^ Key Laboratory of Receptors-Mediated Gene Regulation and Drug Discovery, School of Medicine, Henan University, Kaifeng, Henan, P. R. China

^3^ Key Laboratory of Molecular Pathology, School of basic medical science, Inner Mongolia Medical University, Hohhot, Inner Mongolia, P. R. China

^4^ Department of Materials Science and Engineering, State University of New York at Stony Brook, Stony Brook, New York 11794-2275

^5^ Department of Diabetes and Metabolic Diseases Research, Beckman Research Institute, City of Hope National Medical Center, Duarte, California, 91010


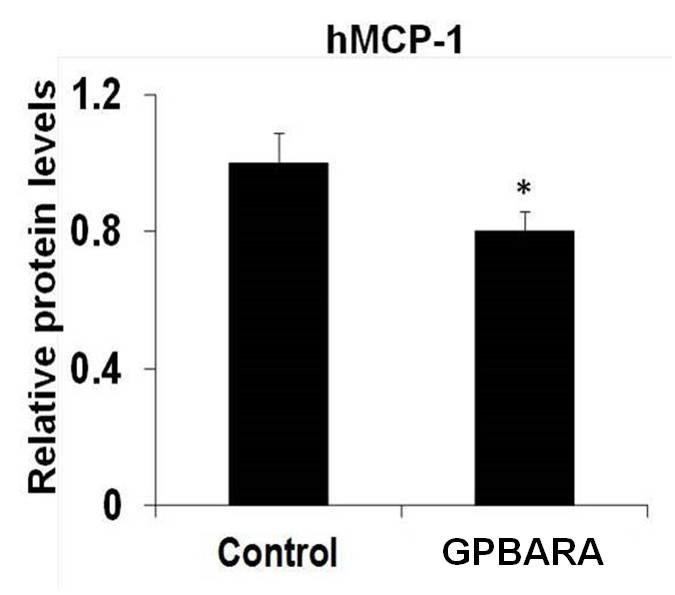


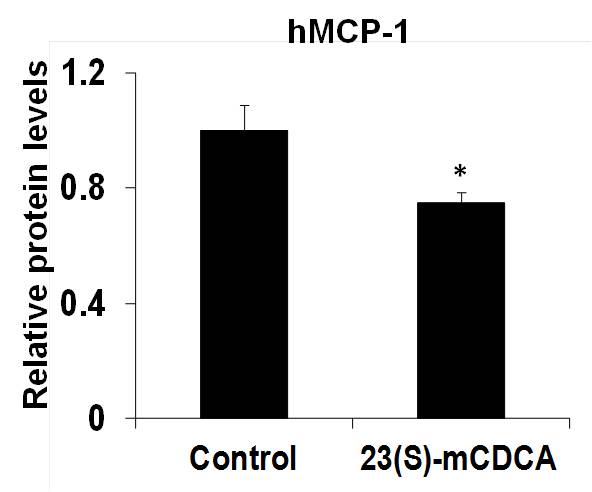

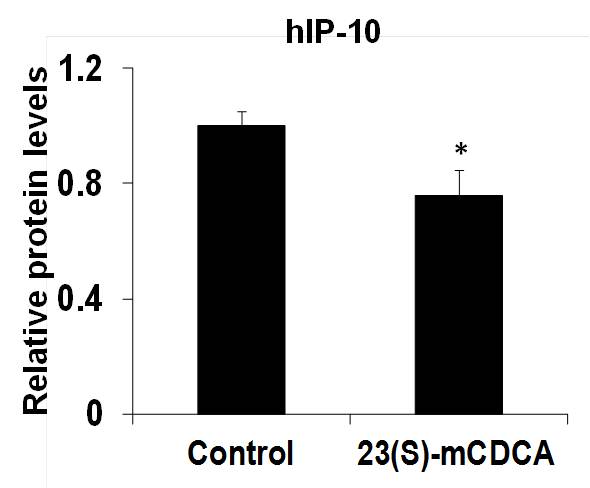

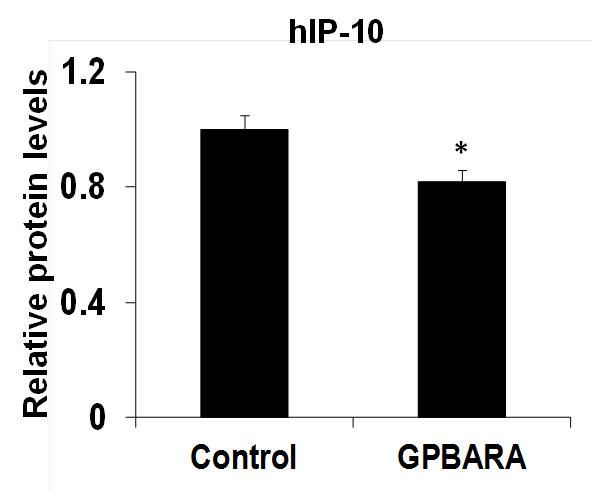


**A**

**B**


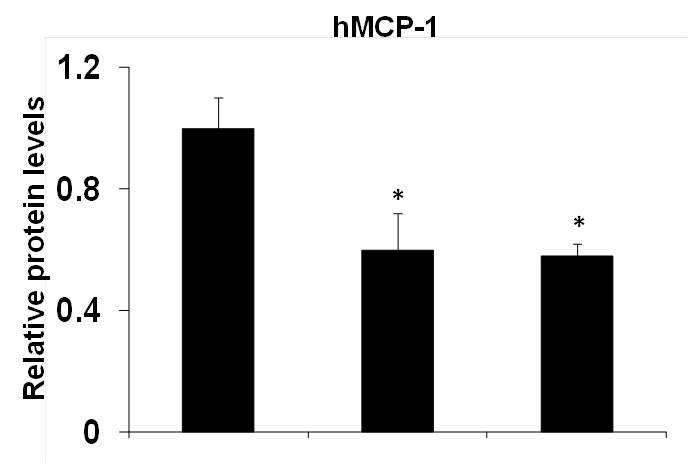


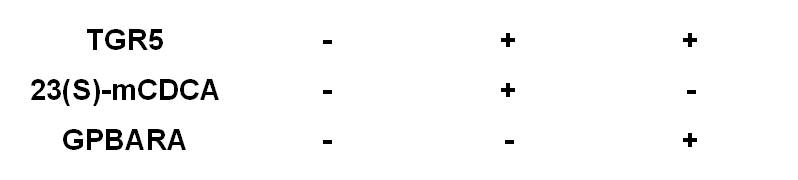


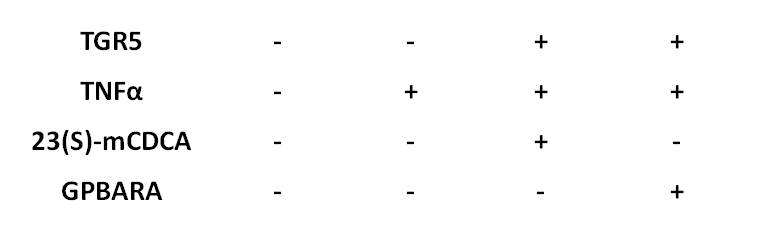

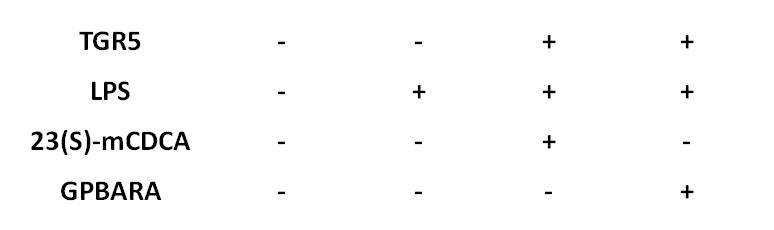

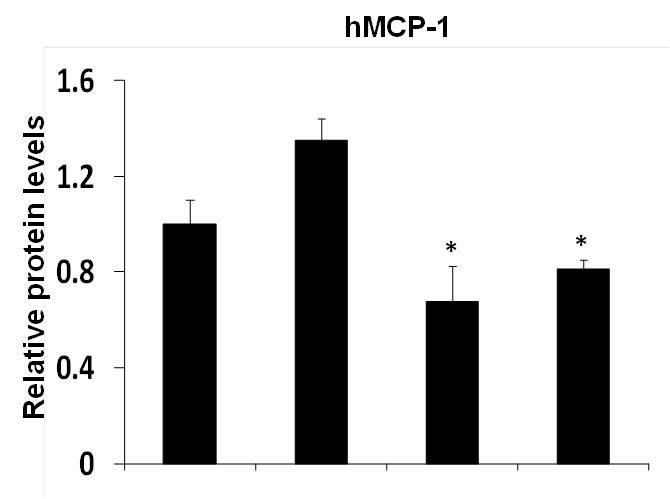

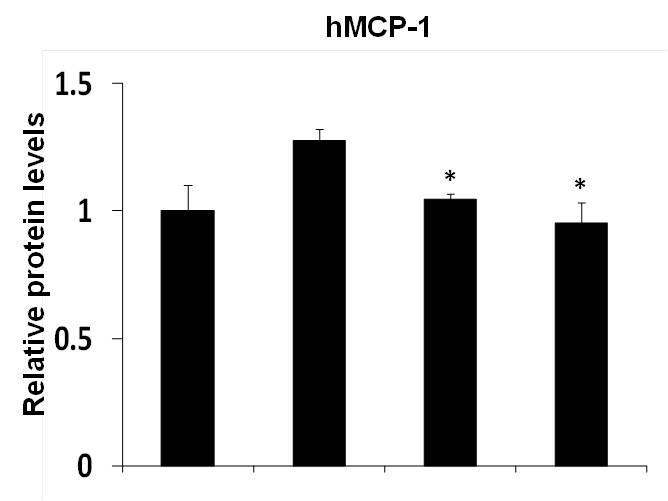


**C**

**Figure S1. Activation of TGR5 suppresses MCP-1 and IP-10 protein expression in gastric cancer cells.** (A) TGR5 ligand treatment suppresses IP-10 and MCP-1 protein expression. 23(S)-mCDCA and GPBARA treated SGC7901 cells for 24 hours. (B) TGR5 overexpression with ligand treatment suppresses MCP-1 protein expression. SGC7901 cells were transfected with the TGR5 expression plasmid or control plasmid. After transfection, cells were treated with GPBARA (3 μM), 23(S)-mCDCA (10 μM) or vehicle (DMSO) for 48 hours. (C) TGR5 activation suppresses LPS or TNF-α-induced MCP-1 protein expression. SGC7901 cells were transfected with the TGR5 expression plasmid or control plasmid. After transfection, cells were treated with GPBARA (3 μM) or vehicle (DMSO) for 48 hours. Before cells were collected, they were treated with LPS (10μg/mL) for 24 hours or TNF-α (10μg/mL) for 6 hour. **P* < 0.05, ***P* < 0.005 versus the control group. (n = 3). The protein levels were tested using ELISA kit from Cloud-clone Corp. (See Materials and Methods).

**A**

**TNF-α**


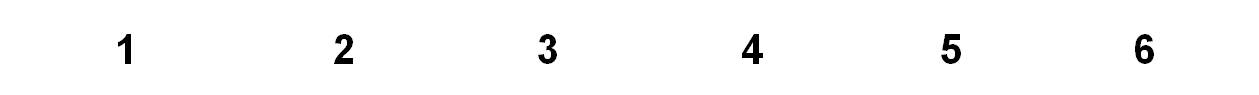

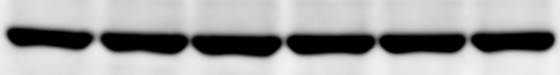

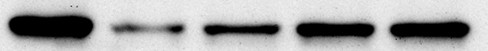

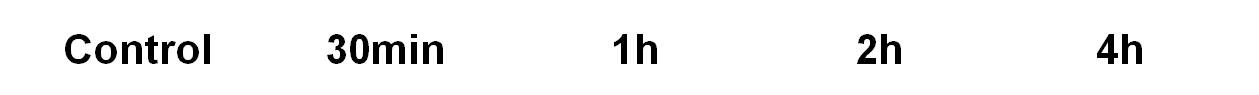

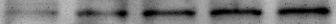


**β-actin**

**T-IκBα**

**P-IκBα**

**B**


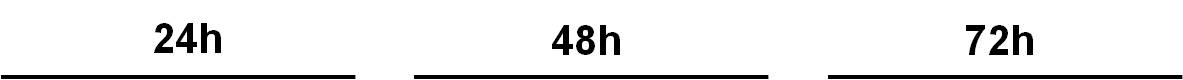


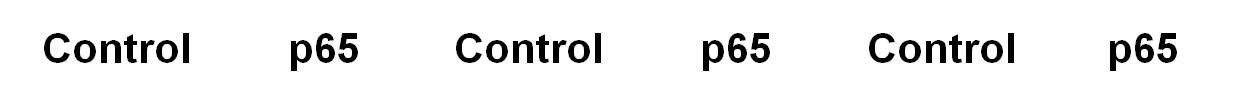


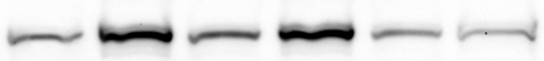


**Nuclear p65 N.P.**


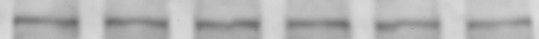


**Lamin B1**


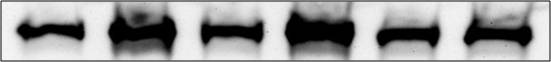


**Cytoplasmic p65**


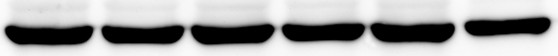


**β-actin**


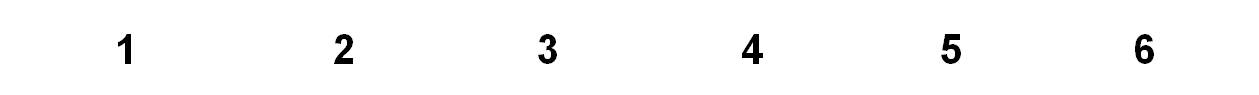


**Figure S2. Activation of IκBα and p65 translocation.** (A) SGC7901 cells were seeded. After 72 hours, cells were treated with TNF-α (10μg/mL) for 30 min, 1, 2 and 4 hours. Finally, cells were collected for western blot analysis. (B) SGC7901 cells were seeded. After 24 hours, cells were transfected with p65 expression plasmid. After 24, 48 and 72 hours of transfection, cells were collected for nuclear and cytoplasmic protein isolation and western blot analysis.
